# Supplementary figures and images for: A Mouse Model Suggests That Heart Failure and Its Common Comorbidity Sleep Fragmentation Have No Synergistic Impacts on the Gut Microbiome
Source: Microorganisms. 2021 Mar 19;9(3):641. doi: 10.3390/microorganisms9030641 (PMC8003359; doi:10.3390/microorganisms9030641)

Species

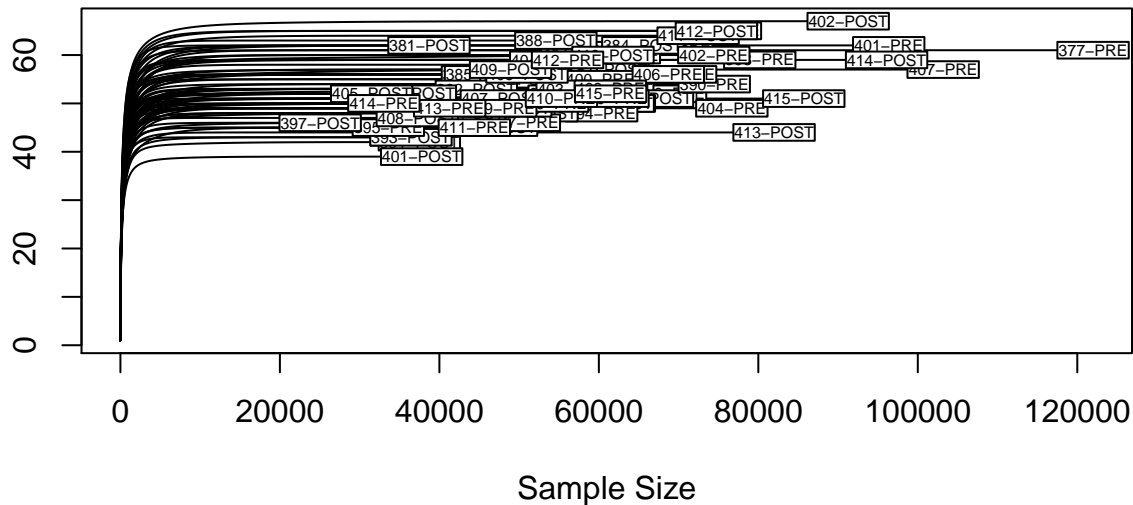

Supplement: Supplementary file 1 [file microorganisms-09-00641-s001.zip › Supplementary/Figure_S1.pdf]

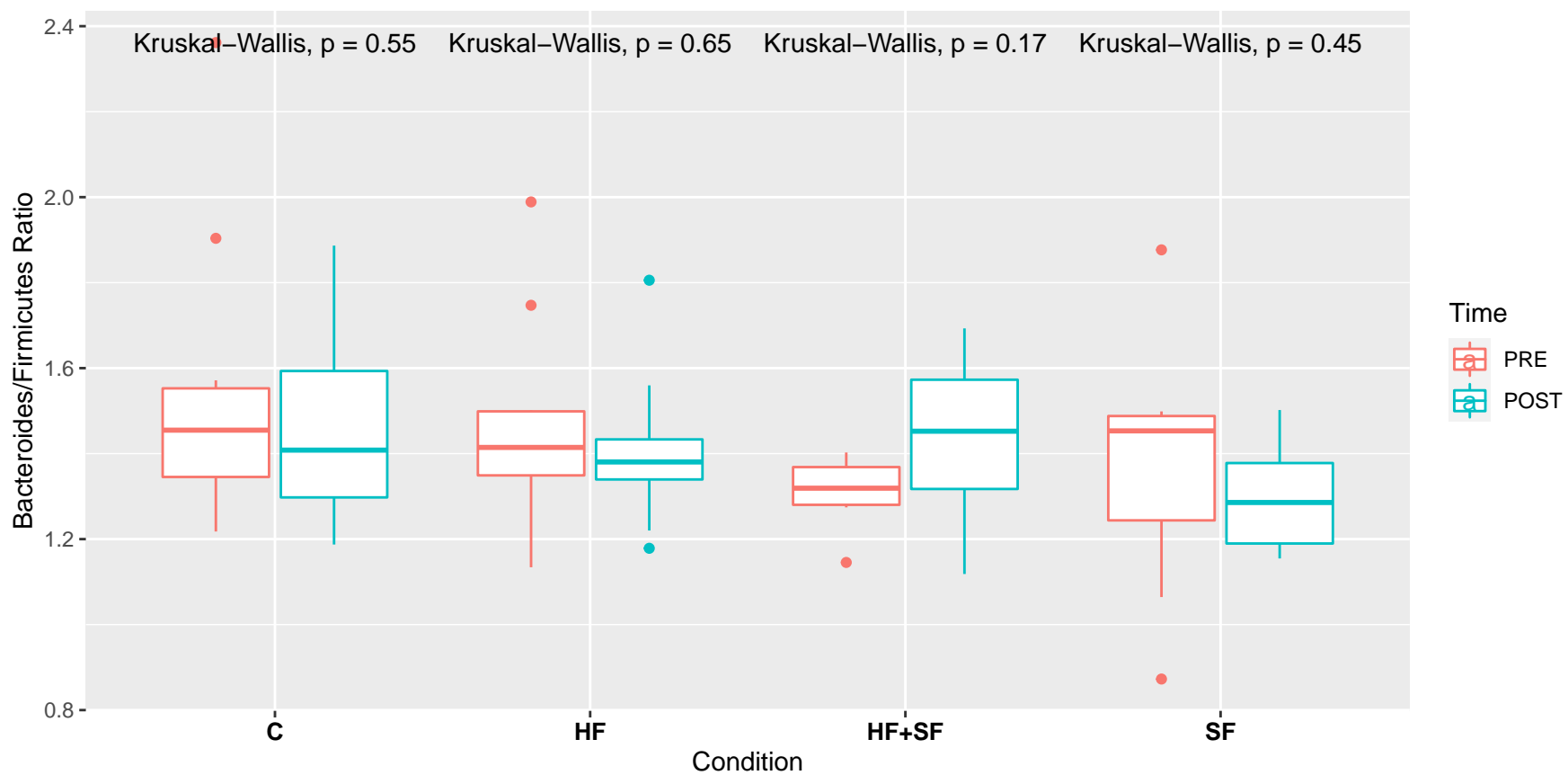

Supplement: Supplementary file 1 [file microorganisms-09-00641-s001.zip › Supplementary/Figure_S2.pdf]
